# Sex‐specific repeatabilities and effects of relatedness and mating status on copulation duration in an acridid grasshopper

**DOI:** 10.1002/ece3.2937

**Published:** 2017-04-04

**Authors:** Michael Haneke‐Reinders, Klaus Reinhold, Tim Schmoll

**Affiliations:** ^1^ Evolutionary BiologyBielefeld UniversityNorth Rhine‐WestphaliaGermany

**Keywords:** copulation duration, inbreeding, mating history, phenotypic control, repeatability, sperm competition

## Abstract

In species with direct sperm transfer, copulation duration is a crucial trait that may affect male and female reproductive success and that may vary with the quality of the mating partner. Furthermore, traits such as copulation duration represent the outcome of behavioral interactions between the sexes, for which it is important—but often difficult—to determine which sex is in phenotypic control. Using a double‐mating protocol, we compared copulation durations between (1) virgin and nonvirgin and (2) sibling and nonsibling mating pairs in rufous grasshoppers *Gomphocerippus rufus*. Nonvirgin copulations took on average approximately 30% longer than virgin copulations, whereas relatedness of mating partners was not a significant predictor of copulation duration. Longer nonvirgin copulations may represent a male adaptation to sperm competition if longer copulations allow more sperm to be transferred or function as postinsemination mate guarding. The absence of differences between pairs with different degrees of relatedness suggests no precopulatory or preinsemination inbreeding avoidance mechanism has evolved in this species, perhaps because there is no inbreeding depression in this species, or because inbreeding avoidance occurs after copulation. Controlling for the effects of male and female mating status (virgin vs. nonvirgin) and relatedness (sibling vs. nonsibling), we found significant repeatabilities (*R*) in copulation duration for males (*R* = 0.33; 95% CI: 0.09–0.55) but not for females (*R* = 0.09; 95% CI: 0.00–0.30). Thus, copulation durations of males more strongly represent a nontransient trait expressed in a consistent manner with different mating partners, suggesting that some aspect of the male phenotype may determine copulation duration in this species. However, overlapping confidence intervals for our sex‐specific repeatability estimates indicate that higher sampling effort is required for conclusive evidence.

## 1 Introduction

Copulation duration in species with direct sperm transfer is an important trait potentially affecting reproductive success in both sexes, in particular in mating systems characterized by sperm competition. In general, copulations are costly due to various reasons, for example, the energetic and time investment in preceding courtship, risk of predation, and possible disease transmission (Daly, 1978; Knell & Webberley, 1999). Potentially, copulation duration is an important factor in determining the magnitude of these costs, because with longer duration energetic costs, predation risk and in addition the costs of missed mating and foraging opportunities likely increase. Brief matings should therefore be favored in both sexes by natural selection but wide variation is exhibited across species and copulation duration can range between few minutes and several days in the same species (see Choe & Crespi, 1997 for examples and an overview).

Many factors can influence copulation duration. One factor could be the degree of relatedness between the mating partners as already described for example in *Drosophila subobscura* (Lizé, McKay, & Lewis, 2014). Because of inbreeding depression (Charlesworth & Willis, 2009), it should be adaptive to avoid matings with close relatives or to reduce the negative effects of these matings, such as decreased number of eggs laid, lower hatching success, or reduced offspring survival (see Keller & Waller, 2002 for an overview). However, when it comes to mating, the risk of inbreeding depression could be reduced or even avoided by limitation or avoidance of sperm transfer. One mechanism to limit the transfer of spermatozoa is the reduction in copulation duration, as these have been shown to be positively correlated (see e.g., Bonduriansky, 2001; Dickinson, 1986; Elgar, Champion de Crespigny, & Ramamurthy, 2003; Engqvist & Sauer, 2003; Lew & Ball, 1980; Parker, Simmons, Stockley, McChristie, & Charnov, 1999; Schneider, Herberstein, Crespigny, Ramamurthy, & Elgar, 2000). From a females’ perspective, this mechanism would reduce the risk of a high parental investment in possibly genetically inferior offspring due to inbreeding depression.

In our study organism, the acridid grasshopper *Gomphocerippus rufus*, nothing is known about potential inbreeding depression. However, inbreeding depression has been found in other species of the order Orthoptera (e.g., Drayton, Hunt, Brooks, & Jennions, 2007; Roff, 1998; Simmons, 2011) and some other studies have shown that crickets evolved kin recognition and inbreeding avoidance mechanisms (Bretman, Newcombe, & Tregenza, 2009; Simmons, 1989; Tuni, Beveridge, & Simmons, 2013). If kin recognition is present in our species and mating between close relatives occurs, should we also expect shorter copulation duration in related pairs than in unrelated pairs?

Aside from the degree of relatedness, also the fact that females of many species copulate with different males within the same reproductive period (Arnqvist & Nilsson, 2000; Birkhead, 2000; Parker, 1970) could be one important factor that influences copulation duration. Postcopulatory sexual selection in the form of sperm competition (Parker, 1970) or cryptic female choice (Eberhard, 1996; Thornhill, 1983) may select for traits that increase male competitive fertilization success which is especially relevant in any highly promiscuous mating system. For example, ejaculate size is an important predictor for fertilization success (Parker & Pizzari, 2010) and males should transfer more sperm under a high risk of sperm competition (but not necessarily under a high intensity of sperm competition Engqvist & Reinhold, 2005). One means to achieve benefits under such circumstances is extending the copulation duration when prolonged copulations lead to the transfer of extra gametes and/or extra nongametic components of the ejaculate that promote competitive fertilization success (e.g., elevating the current fecundity of the female in *Drosophila,* Chapman, Liddle, Kalb, Wolfner, & Partridge, 1995). Additionally, courtship signals produced during copulation could lead to an increased fertilization success due to cryptic female choice and may extend copulation duration (Eberhard, 1996). Furthermore, pre‐ or postinsemination mate guarding or the removal of an ejaculate from another male could provide a mechanistic basis for the benefits of longer copulations (e.g., Jarrige, Kassis, Schmoll, & Goubault, 2016 and see Alcock, 1994; Danielsson, 1998 and Simmons, 2001 for an overview). For these traits, selection is probably stronger in males because their fitness is determined by their fertilization success, whereas female fitness is usually mainly limited by the ability to require resources and convert them into eggs.

Several studies show that males adjust their copulation duration in response to sperm competition in different insect species (e.g., Andrés & Cordero Rivera, 2000; Bretman, Fricke, Hetherington, Stone, & Chapman, 2010; Bretman, Fricke & Chapman, 2009) and also in other taxa (see Kelly & Jennions, 2011 for an overview). If males are capable of sensing the mating status of a female partner, they should copulate longer with nonvirgin females as a response to the higher perceived sperm competition risk. Indeed, a number of studies have shown the male ability to recognize the mating status of the female in different insects (e.g., Carazo, Sanchez, Font, & Desfilis, 2004; King & Dickenson, 2008; Siva‐Jothy & Stutt, 2003; Yamane & Yasuda, 2014) and longer copulation durations in matings with nonvirgin females (e.g., Andrés & Cordero Rivera, 2000; Friberg, 2006; Wedell, 1992).

Sperm competition is also described for grasshopper species of the family Acrididae and therefore for closely related species of our study organism. For example, it is known that females of *Chorthippus parallelus* copulate several times with different males, both in the laboratory (e.g., Bella, Butlin, Ferris, & Hewitt, 1992; Haskell, 1958; Reinhardt & Köhler, 1999; Ritchie, Butlin, & Hewitt, 1989) and importantly also in field (Reinhardt, Köhler, Webb, & Childs, 2007). Reinhardt (2000) also showed that after two copulations, the sperm precedence pattern (measured as P_2_‐value, indicating the relative fertilization success for the second of two males) varies between two closely related acridid species (*Chorthippus parallelus* and *C. biguttulus*) and even within a single population (in *C. parallelus*). In general, P_2_‐values of different Acridoidea grasshopper species differ between and within the species but the mean value is mostly above 0.5, which means the second male has an fertilization advantage (see Table 2.3 in Simmons, 2001 for an overview). Female mating rates and sperm precedence patterns have not been examined in natural populations of our model organism, *G. rufus*, but females copulate multiple times with different males in the laboratory (e.g., Hartmann & Loher, 1999; Loher & Huber, 1964; Riede, 1983), suggesting sperm competition has been an important selection pressure also in this species.

For traits involved in behavioral interactions between the sexes during mating, it may be difficult to determine which sex is in phenotypic control of the respective behavior. For example, copulation duration can only be attributed to a pair of mating partners but likely is determined to a large part by just one of the partners. Thus, we here also assessed sex‐specific repeatabilities in copulation durations obtained from a double‐mating experimental design to test whether copulation durations of individual male and/or female grasshoppers would represent a nontransitive trait expressed in a consistent manner against different female or male partner backgrounds.

Here, we present copulation duration data measured in a double‐mating experiment. Our study species needs only a few minutes to produce and transfer a spermatophore, yet copulations typically last for an hour or more (Hartmann, 1970). The experimental design used in this experiment allowed us to address three questions: (1) Does copulation duration differ between sibling pairs and nonsibling pairs? (2) Does copulation duration differ between nonvirgin and virgin copulations? (3) Which sex is in phenotypic control of copulation duration?

## 2 Materials and Methods

### 2.1 Study species and experimental animals

*Gomphocerippus rufus* L. is an acridid grasshopper that is distributed widely across Europe and parts of Asia (Bisby et al., 2012; Eades, Otte, Cigliano, & Braun, 2012) and preferentially inhabits semidry meadows, shrubby areas, and forest margins. Females lay egg pods containing eight to ten eggs into the soil (Loher & Huber, 1964) and the offspring hatch in the subsequent spring after overwintering. Females can lay two to three egg pods per week and adults survive for about eight to twelve weeks under laboratory conditions (Hartmann & Loher, 1999).

For experiments, we used the F1 generation bred from wild animals which were collected as nymphs (in the last two nymphal stages, L3‐L4) in August 2013 in Tübingen, Germany (48°30.1′N; 9°3.9′E). The F0 nymphs were separated by sex after capture and 2 or 3 days after final ecdysis, F0 females were mated at random to a single F0 male and subsequently kept separately in plastic cages where they were allowed to lay eggs in sand pots (diameter 4 cm). The sand was sieved for egg pods every week, and the egg pods were collected and kept in Petri dishes lined with moist filter paper. Petri dishes were stored for 4–6 weeks at room temperature and were then transferred to 4–6°C for at least 2 months to simulate the hibernating phase. After hibernation eggs were kept in a heated room at approximately 28°C (daytime) or 20°C (at night) with a 14:10 hr light:dark cycle where F1 larvae hatched after 1–3 weeks. These F1 offspring were kept family‐wise in cages under identical temperature and light conditions. After final ecdysis, animals were marked individually on the day of ecdysis and separated by sex to ensure virginity. We provided all animals with a mix of grasses (Poaceae) ad libitum.

### 2.2 Staging of experimental matings

**Figure 1:**
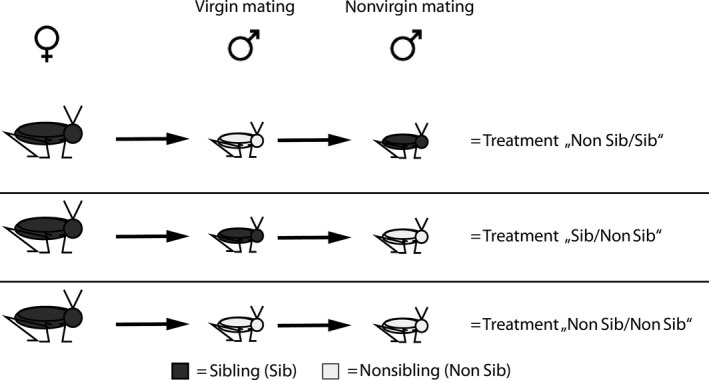
Experimental design: Mating scheme for females’ treatment. Every female was mated twice with different males. The color coding gives information about the degree of relatedness within the mating triplet. Gray filled females are full‐siblings to gray filled males but not to not filled (white) males. In combination with mating order the treatment levels Non Sib/Sib, Sib/Non Sib and Non Sib/Non Sib result. Also most males were used twice in a similar way (e.g., first in a sibling mating second in a nonsibling mating). A scheme for males’ matings would look equal

For the purpose of investigating the effects of mate relatedness on copulation duration as well as on further postcopulatory inbreeding avoidance mechanisms, we used a double‐mating protocol involving three groups: In the first group, we offered a nonsibling (Non Sib) virgin male as mating partner for a virgin female's first copulation and a full sib (Sib) nonvirgin male for that female's second copulation. In the other groups, the respective sequence was Sib/Non Sib and Non Sib/Non Sib with the latter acting as our control group (see Figure 1). Because of the low copulation rate in nonvirgin matings, not all males which successfully copulated in virgin matings engaged in nonvirgin copulations.

Females were seven to 14 days old (mean: 9.6, SD: 1.41) at virgin mating, and males were between four and 18 days old (mean: 9.7, SD: 2.31). After natural termination of virgin copulations, animals were kept in isolation before staging nonvirgin matings. Females experienced a nonmating period of at least seven up to nine and males of at least five up to 10 days between the two experimental matings. This is necessary due to the secondary defense behavior in this species, in which a female successfully avoid copulation by kicking males with the hindlegs and which is induced by secretion of first males’ spermatophore. With this interval between experimental matings, we ensured that females had had sufficient time to lay the first egg pods, which usually terminates the secondary defense phase and means the females are receptive to remating (Hartmann & Loher, 1996, 1999). Males experienced a similar mating interval for purely logistical reasons and to keep the age comparable between females and males. All pairs were placed in a plastic cage (15 cm × 15 cm × 20 cm) under artificial light at 28°C and were monitored for mating activity. Pairs not starting copulation within 180 min were discarded. We measured copulation durations (range from 17 to 213 min) to a precision of 1 min using stopwatches. To minimize observer bias, the observer did not know the treatment group to which each mating pair belonged. In most cases, it was also unknown to the observer whether a virgin or a nonvirgin copulation was being observed (except for the last copulations of the experiment).

### 2.3 Experimental design and statistical analysis

In addition to testing for the effects of relatedness on mate choice, our experiment was also designed to explore the potential for postcopulatory inbreeding avoidance via cryptic female choice, through the analysis of paternity allocation in relation to experimentally manipulated relatedness. Because of different mortality rates and a remating rate of around 60%, not all individuals engaged in nonvirgin copulations resulting in a total of 143 virgin (of 196 females in total) and 72 nonvirgin copulations of females. The higher mortality in males was also the reason why we had copulations in which the mating statuses of females differed from those of males. There were twelve mating pairs with nonvirgin females and virgin males and two mating pairs with virgin females and nonvirgin males. We checked whether these copulations had any influence on our model fits (see Appendix S1 for results with the full dataset) and as the effects were weak, we excluded them from further analyses resulting in 141 virgin (96 copulations with nonsiblings and 45 with siblings) and 60 nonvirgin copulations (24 for Non Sib/Non Sib, 17 for Non Sib/Sib and 19 for Sib/Non Sib group).

We tested for systematic differences in copulation duration between sibling and nonsibling matings with a linear mixed effects model using the R function *lmer* from the *lme4* package (Bates et al. 2015). This model included the relatedness (two levels: sibling or nonsibling) and male/female mating status (two levels: virgin or nonvirgin) as fixed effects. Male and female identities were fitted as random effects to account for repeated sampling of the same individuals. Because of the strong correlation of age and mating status in both sexes, we did not include male or female age in the model to avoid collinearity of predictors. Significance of fixed and random effects was determined by removing the focal term from the current model using ML fits when testing for fixed and REML fits when testing for random effects. *p*‐values refer to the increase in model deviance when the relevant term was removed compared against a χ^2^ distribution using likelihood ratio tests (LRT).

To test whether mating order had a significant influence, we analyzed the durations of nonvirgin copulations between the three different treatment groups (see section 2.2). We used a linear model (one‐way ANOVA) with treatment group fitted as a three‐level categorical fixed factor (Non Sib/Sib, Sib/Non Sib and Non Sib/Non Sib). We did this separately for both sexes as treatments sometimes differed between male and female mating partners.

After these analyses and the finding that the mating status has a significant influence on copulation duration (see results), we used within‐subject paired *t* tests based on the subsamples of females or males that were successfully sampled twice (*N* = 59 and *N* = 58, respectively) to test for systematic differences in copulation durations between virgin and nonvirgin matings of the same individual. The deviation from the above‐mentioned 60 nonvirgin copulations occurs because we excluded 14 copulations from this analysis in which the mating status of female and male did not match (e.g., one female copulated with a nonvirgin male in its own virgin copulation and two males copulated with nonvirgin females in their own virgin copulations).

Furthermore, we used the linear mixed effects model described above to calculate repeatabilities of copulation durations across virgin and nonvirgin matings of the same individual. Contingent on the fixed effects and based on a restricted maximum likelihood (REML) fit of the model, the repeatability of copulation duration for males was calculated as the between‐male variance divided by the total variance in copulation duration. Based on variance estimates from the same model fit, the repeatability of copulation durations for females was likewise calculated as the between‐female variance divided by the total variance in copulation duration. To obtain 95% confidence intervals for repeatability estimates, we carried out parametric bootstrapping with 10,000 replicates following Faraway (2006) and Nakagawa and Schielzeth (2010). To determine the significance of random effects in this model, we removed the focal term from a REML fit of the model. The corresponding *p*‐value refers to the observed increase in model deviance (compared against a χ^2^ distribution) when a focal term is removed from the model. All statistical analyses were performed in R 3.1.3 (R Core Team, 2015).

## 3 Results

### 3.1 Copulation duration in relation to relatedness and mating status

**Figure 2:**
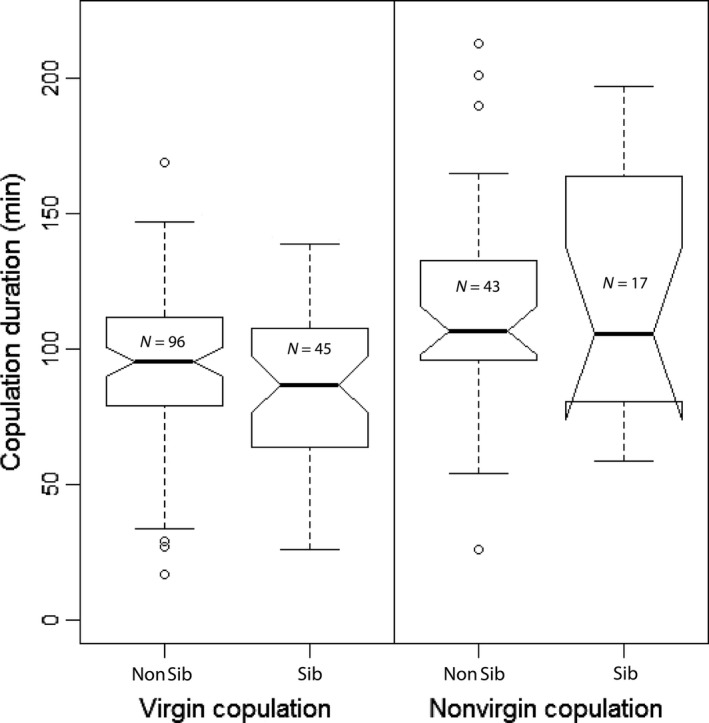
Durations of virgin and nonvirgin copulations with full‐siblings (Sib) or nonsiblings (Non Sib). No differences in copulation duration between siblings or nonsibling pairs were found neither in virgin nor in nonvirgin copulations. (Standard notched boxplot. Notches show 95% CI of the median, whiskers show upper and lower quartile of the data, circles represent outliers)

There were no significant differences between sibling and nonsibling pairs overall (Table 1). When analyzed separately for virgin and nonvirgin copulations, the sibling status showed also no effect (Figure 2, virgin copulations: *F*_1,139_ = 2.83, *p* = .09; nonvirgin copulations: *F*_1,58_ = 0.36, *p* = .55, linear model fits). Additionally, copulation probability was not affected by the degree of relatedness (virgin matings: *F*_1,194_ = 0.11, *p* = .74; nonvirgin matings: *F*_1,113_ = 0.45, *p* = .51, linear model fits). However, we found a highly significant effect of mating status on copulation duration, with longer copulations in nonvirgin as compared to virgin pairs (Table 1).

**Table 1:** Linear mixed effects model fit for copulation duration of *G. rufus* treating relatedness and mating status as two factorial predictors with two levels each

Fixed effects	Estimate	SE	χ²	*p*
Intercept	119.10	4.30		
Relatedness (sibling)	−4.31	4.65	0.87	.35
Pair copulation type (virgin)	−25.94	4.25	29.35	<.001

Random effects	Variance	SD	χ²	*p*
Female ID (intercept)	65.97	8.12	0.36	.55
Male ID (intercept)	351.00	18.74	8.83	<.003

**Figure 3:**
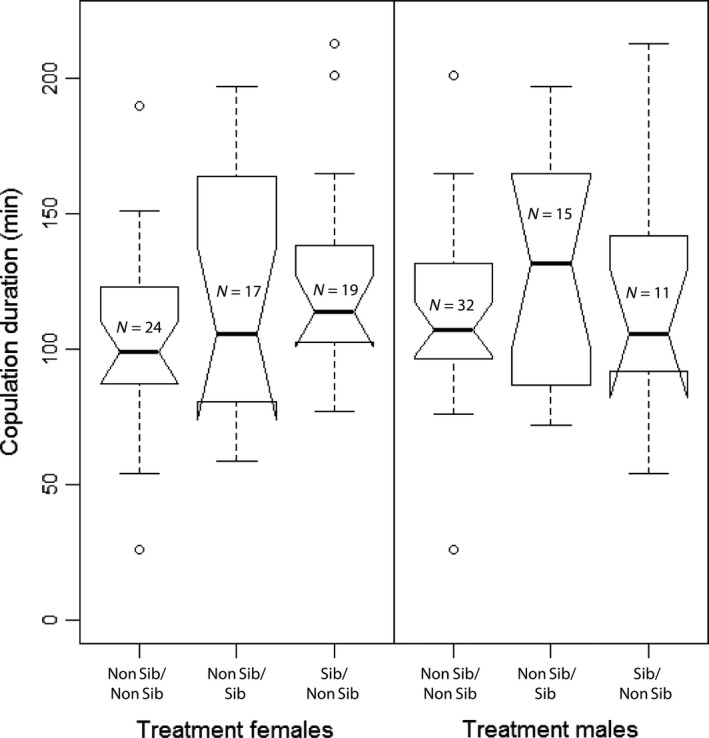
Duration of nonvirgin copulations sorted by sex and treatment. Only nonvirgin copulations are shown to visualize that the treatment (mating order) had no influence on duration. Because of the twofold use of females and males the treatment differs between the sexes. The sample size also differs between the sexes because not all males were used successfully twice (60 females vs. 58 males, see also section 2). Treatment levels: Non Sib/Non Sib = first and second copulation with nonsibling; Non Sib/Sib = first copulation with nonsibling, second with sibling; Sib/Non Sib = first copulation with sibling, second with nonsibling. (Standard notched boxplot. Notches show 95% CI of the median, whiskers show upper and lower quartile of the data, circles represent outliers)

Comparing nonvirgin copulations separately for females and males, we did not find significant differences between treatments in copulation duration (Figure 3). The copulation order (first sibling or nonsibling and second sibling or nonsibling, i.e., the treatment) had no influence on the copulation duration in the second copulation in both sexes (linear model fits, females: *F*_2,57_ = 1.94, *p* = .15; males: *F*_2,55_ = 0.94, *p* = .40, comparing of the three treatment groups as in Figure 3).

### 3.2 Within‐individual effects of mating status

**Figure 4:**
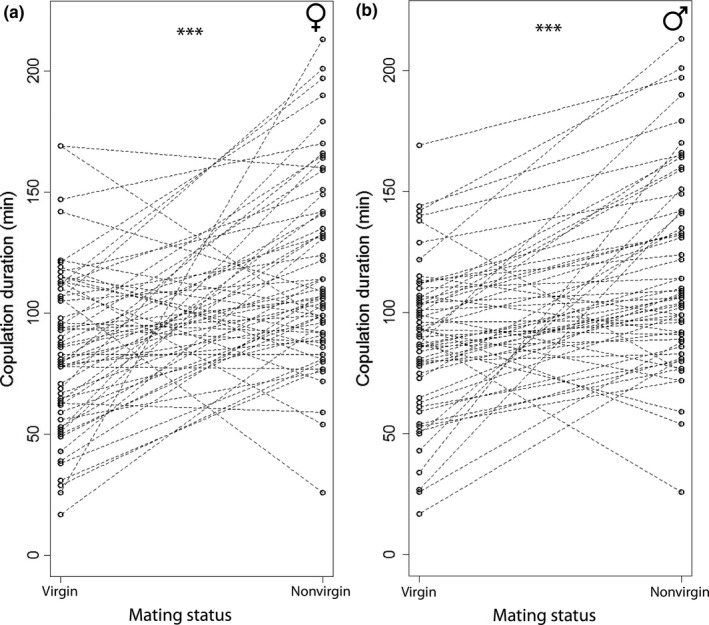
Copulation duration in relation to sex‐specific mating status for (a) *N* = 59 females and (b) *N* = 58 males. Lines connect virgin and nonvirgin copulations of the same individuals (paired *t* tests: females *t*_58_ = 5.01, *p* < .001; males *t*_57_ = 5.39, *p* < .001)

We analyzed the subsets of individuals that were successfully mated twice to directly model the within‐subject response to mating status. Female nonvirgin copulations were on average 31.7 (95% CI: 19.0–44.4) minutes longer compared to corresponding virgin copulations (paired *t* test: *t*_58_ = 5.00, *p* < .001, Figure 4a, Table 2). Likewise, male nonvirgin copulations were on average 27.9 (95% CI: 17.5–38.2) minutes longer than corresponding virgin copulations (paired *t* test: *t*_57_ = 5.39, *p* < .001, Figure 4b, Table 2).

**Table 2:** Summary statistics for copulation durations (min) separated by sex and copulation type (regardless of mating partner relatedness) and paired *t* test testing for differences between mating status within sexes

Sex	Copulation type	Mean ± *SD*	Range	*N*	*p*
Females	Virgin	85.0 ± 33.3	17–169	59	<.001
	Nonvirgin	116.7 ± 38.6	26–213		
Males	Virgin	88.7 ± 30.3	17–169	58	<.001
	Nonvirgin	116.6 ± 39.2	26–213		

### 3.3 Sex‐specific repeatabilities of copulation duration

Repeatabilities for copulation duration amounted to 0.33 (95% CI: 0.09–0.55, LRT: χ^2^ = 8.83, *df* = 1, *p* < .003) for males and to 0.09 (95% CI: 0.00–0.30, LRT: χ^2^ = 0.36, *df* = 1, *p* = .55) for females.

## 4 Discussion

Copulation durations and copulation probability do not differ between siblings and nonsibling pairs in *G. rufus*. But we found around 30% longer copulation in nonvirgin pairs compared to virgin pairs. In the following paragraphs, we discuss possible reasons for the lack of differences between copulations with siblings (inbreeding) and nonsiblings. Afterward, we focus on the longer nonvirgin copulation and discuss sperm competition as the probably most likely reason for that. In the end, we discuss the result of the repeatability analysis and in which sex the selection pressure should be higher to determine the copulation duration.

Despite the risk of inbreeding depression, we found no detectable discrimination of siblings either by females or males. If there is only weak or even no inbreeding depression in this species, evolving an inbreeding avoidance mechanism might be too costly or unnecessary. In addition, inbreeding can also have advantages for both partners because of the increased inclusive fitness by mating with close relatives (Parker, 1979). Indeed, matings with close relatives are regularly observed in nature (Keller & Waller, 2002). The optimal level of inbreeding that maximizes inclusive fitness depends on the strengths of inbreeding depression (Puurtinen, 2011) and on the costs of inbreeding avoidance versus the benefits of mating with kin (Kokko & Ots, 2006). Unfortunately, information on inbreeding depression in our study organism is currently lacking.

Another point to consider is that in species where males do not invest more than their (comparatively cheap) ejaculate, rejecting matings is likely much more costly for males than matings with close relatives. Thus, males of these species should be selected to realize similar copulation durations with related and unrelated females. If the related female is nonvirgin, the male should copulate even longer with it than with an unrelated virgin female because of the sperm competition risk (as discussed in the next paragraphs). Furthermore, females should be selected to avoid inbreeding because of the higher parental investment whereas males should probably be selected to inbreed to increase their inclusive fitness (Facon, Ravigné, & Goudet, 2006; Kokko & Ots, 2006; Parker, 1979; Pizzari, Lo, & Cornwallis, 2004). In other words, if males control copulation duration, we would not expect differences between sibling and nonsibling pairs, which is the pattern we find in our data.

However, a more simple explanation for our findings could be that there is no kin recognition in our species. In fact, there are no studies about kin recognition in *G. rufus* and also our data cannot answer the question whether this species is capable to recognize close relatives like siblings. In addition, we are not aware of a study of kin recognition in acridid grasshoppers at all. At least there are some studies that show species recognition and also mate recognition in *G. rufus* (e.g., Jacobs, 1950; Loher & Huber, 1964; Riede, 1983). In general, species and sex recognition by the song is described for other close related species of *G. rufus* (e.g., see Balakrishnan, von Helversen, & von Helversen, 2001; von Helversen, 1972; von Helversen & von Helversen, 1997; Klappert & Reinhold, 2003; Safi, Heinzle, & Reinhold, 2006 for *Chorthippus biguttulus*, Charalambous, Butlin, & Hewitt, 1994; Perdeck, 1958; Saldamando et al., 2005 for *C. brunneus* and Stumpner & von Helversen, 1994 for examples in three other species of the *Chorthippus* genus) and in the case of *C. biguttulus* and *C. mollis* also by chemical cues (Finck, Kuntze, & Ronacher, 2016). Additionally, Ritchie et al. (1989; see also Butlin & Ritchie, 1991) demonstrated assortative mating across a hybrid zone in the close related species *Chorthippus parallelus*. They showed that matings in pairs of the same subspecies (*C. p. parallelus* and *C. p. erythropus*) are more likely than between subspecies, which differ by their songs (Ritchie, 1990). However, there is gene flow between the subspecies and hybrids occur (Bella, Serrano, Orellana, & Mason, 2007; Bella et al., 1992), the upper mentioned mating experiments showed mate recognition and a preference for mates of the same subspecies. Because of the upper cited studies, we have reasons for the assumption that *G. rufus* is capable to recognize at least other members of the same species (species recognition) as well as possible mating partners (sex recognition). To clarify whether there is kin recognition in this species, more or other experiments are necessary but it will be still difficult if *G. rufus* does not discriminate between relatives at all.

Our results also demonstrate more than 30% longer copulation durations in nonvirgin compared to virgin matings. Such prolonged nonvirgin copulations have been shown for a number of insect species (e.g., in a beetle (Dickinson, 1986), a bush‐cricket (Wedell, 1992), a damselfly (Andrés & Cordero Rivera, 2000), a dung fly (Martin & Hosken, 2002), two different bug species (García‐González & Gomendio, 2004; Siva‐Jothy & Stutt, 2003), *Drosophila melanogaster* (Friberg, 2006) (but see Singh & Singh, 2004 for the opposite in other *Drosophila* species) and in the lesser wax moth *Achroia grisella* (Engqvist, Cordes, Schwenniger, Bakhtina, & Schmoll, 2014)). But we are not aware of studies that describe different copulation duration between virgin and nonvirgin copulations in acridid grasshoppers. Under the assumption that males can sense female mating status, prolonged nonvirgin copulation durations may thus represent a male adaptation to sperm competition and several possible mechanisms are conceivable. For example, mechanical sperm removal has been shown in insects (e.g., in dragonflies (Córdoba‐Aguilar & Cordero‐Rivera, 2008; Waage, 1979), in a beetle (Gage, 1992) and in the bush‐cricket *Metaplastes ornatus* (von Helversen & von Helversen, 1991)) which could be time‐consuming and thus explain longer copulation duration in matings with already mated as compared to virgin females. Furthermore, sperm removal by flushing sperm of a previous male from the female sperm storage organ with its own ejaculate (Danielsson, 1998) has been suggested as a possible mechanism in the tree cricket *Truljalia hibinonis* (Ono, Siva‐Jothy, & Kato, 1989). The transfer of extra gametes or a higher volume of seminal fluid for flushing could also need more time. However, it is unknown whether male acridid grasshoppers are capable of removing sperm of competitors.

Further reasons for prolonged copulations may include in‐copula mate guarding (Alcock, 1994) or the possibility to transfer a larger ejaculate, which has been shown for the acridid grasshoppers *Dichromorpha viridis* (Johnson & Niedzlek‐Feaver, 1998), *Melanoplus differentialis* (Hinn & Niedzlek‐Feaver, 2001), and the desert locust *Schistocerca gregaria* (Dushimirimana, Hance, & Damiens, 2012; Pickford & Padgham, 1973). Pickford and Padgham (1973) also demonstrated that males of *S. gregaria* regularly transfer not only more spermatozoa but also more than one spermatophore in a single copulation, which needs more time the more spermatophores will be produced and transferred. Two years earlier, Pickford and Gillott (1971) found the same in *Melanoplus sanguinipes* and a correlation between copulation duration and the number of transferred spermatophores. Hinn and Niedzlek‐Feaver (2001) similarly found more than one transferred spermatophore after a single copulation in *M. differentialis*.

Considering the long copulation duration and because Hartmann (1970) clearly showed that *G. rufus* needs only around 4 min to produce and transfer a spermatophore, we cannot exclude that *G. rufus* males are also capable of producing and transferring more than one spermatophore within a single copulation. The transfer of more spermatophores could enhance fertilization success in the presence of sperm competition and another study showed for *L. migratoria* that *P*_2_‐values were significantly higher after longer copulations of the second male (compared to shorter copulations of second males Zhu & Tanaka, 2002; but see Reinhardt & Meister, 2000 for no correlation between ejaculate size and copulation duration in *L. migratoria*). The fact that *G. rufus* males produce the spermatophore only after starting the copulation (see Hartmann, 1970 again) makes an adjustment of the sperm number based on female mating status possible. This would be an advantageous adaptation to sperm competition. However, it is unknown for our study organism how fast males can produce a second or a third spermatophore after the previous one. Perhaps they need a short recovery period between the production of two spermatophores, potentially leading to an extended copulation duration as measured in the nonvirgin copulations.

Furthermore, it is possible that males need more time to transfer gametes and/or to produce spermatophores when they are older. In our experiment, males were 5–10 days older in the nonvirgin mating than in the virgin mating. Alternatively, older females may need more time for processing a spermatophore (females were seven to 14 days older in the nonvirgin matings). However, Wedell (1992) showed that there are no differences in copulation duration between virgin and nonvirgin males in another Orthopteran species, the wartbiter *Decticus verrucivorus* (Orthoptera; Tettigoniidae). Nevertheless, she found differences between virgin and nonvirgin females’ copulation duration and concluded that female mating status could be detected by males. The potential mechanism of mating status recognition in our species is unknown, but some possible ways have been shown for other insect species (see introduction and Thomas, 2011 for an overview). Our results are consistent with female mating status recognition by males in *G. rufus*, but further studies are needed to conclusively test this hypothesis.

Controlling for systematic effects (i.e., mating status and relatedness), sex‐specific repeatabilities of copulation duration (0.33 in males vs. 0.09 in females) suggested that aspects of the male phenotype determine copulation duration, a behavioral interaction trait only displayed by pairs. Numerous studies provided evidence that males of different insect and spider species are in control of the copulation duration (e.g., Bretman, Westmancoat, & Chapman, 2013; Hughes, Siew‐Woon Chang, Wagner, & Pierce, 2000; Mazzi, Kesäniemi, Hoikkala, & Klappert, 2009; Vahed, Lehmann, Gilbert, & Lehmann, 2011; Wilder & Rypstra, 2007), and Engqvist et al. (2014) also found higher repeatability in males than in females for copulation duration in a moth. However, the confidence intervals for male and female repeatabilities overlap and thus higher sampling effort is required for conclusive evidence. Even though the mean values are not affected from these overlaps, we have to interpret our repeatability data with care.

Nevertheless, we also observed several times that during copulation males were kicked by the females and/or lost contact with the female's body with their legs while still in copulation, sometimes for more than 30 min (Haneke‐Reinders, pers. obs.). This suggests that the males but not the females can terminate the connection of the sexual organs and thus the males seem to be in phenotypic control of copulation duration. In light of the findings and studies discussed above, this fits to the expectation that the selection pressure resulting from sperm competition to control the copulation duration should be higher in males.

From a female perspective, however, longer copulations with particular mates could well be beneficial too. The animals in our experiment came from the same population, and it is likely that females of one population react in a similar way to male attractiveness. For example, females of the grasshopper *Chorthippus biguttulus* prefer courtship songs with pauses of a specific duration between the syllables (von Helversen, 1972; Klappert & Reinhold, 2003) and Reinhold, Reinhold, and Jacoby (2002) showed that female responses to courtship songs were repeatable between songs. If heritable male fitness underlies male sexual attractiveness, females could have an advantage when they copulate longer with attractive than with less attractive males assuming an increased copulation duration increases the fertilization success of these males. In such a case, we would also measure a high repeatability for males because females would adjust the copulation duration by the phenotype (the attractiveness) of these males. However, this hypothesis cannot explain the observed differences in copulation duration between virgin and nonvirgin female copulations and it therefore seems more likely that the males are in control.

In conclusion, we found that in our study species, nonvirgin copulations were significantly and substantially longer than virgin copulations. Concerning copulation duration, we also found no evidence in favor of precopulatory or preinseminational inbreeding avoidance in this species. Furthermore, sex‐specific repeatabilities of copulation duration suggest that aspects of the male phenotype determine copulation durations.

## Conflict of interest

None declared.
