# Supplementary material for: Sex‐specific repeatabilities and effects of relatedness and mating status on copulation duration in an acridid grasshopper
Source: Ecol Evol. 2017 Apr 4;7(10):3414–24. doi: 10.1002/ece3.2937 (PMC5433977; doi:10.1002/ece3.2937)
Supplement: Supplementary file 1 [file ECE3-7-3414-s001.docx]

**Electronic Supplement**

**Sex-specific repeatabilities and effects of relatedness and mating status on copulation duration in an acridid grasshopper**

**Michael Haneke-Reinders^1^*, Klaus Reinhold^1^ & Tim Schmoll^1^**

^1^Bielefeld University, Evolutionary Biology, Germany

*Correspondence: M. Haneke-Reinders, Evolutionary Biology, Bielefeld University, Morgenbreede 45, 33615 Bielefeld, Germany

E-mail address: michael.reinders@uni-bielefeld.de

Phone: ++49/521/106 2727

Key Words: copulation duration – inbreeding – sperm competition – mating history – repeatability – phenotypic control

**Results of statistical analyses with the full data set of 215 copulations**

*Copulation duration in relation to relatedness*

There were no significant differences between sibling and non-sibling pairs overall (Table 1). When analysed separately for virgin and non-virgin copulations the sibling status showed no effect neither (virgin copulations: F_1,141_ = 3.072, p = 0.08; non-virgin copulations: F_1,70_ = 0.53, p = 0.47, linear model fits). Additionally, copulation probability was not affected by the degree of relatedness (virgin matings: F1,194 = 0.11, p = 0.74; non-virgin matings: F1,113 = 0.45, p = 0.51, linear model fits). However, we found a highly significant effect of mating status on copulation duration, with longer copulations in non-virgin as compared to virgin pairs (Table 1).

Comparing non-virgin copulations separately for females and males we did not find significant differences between treatments in copulation duration. The copulation order (first sibling or non-sibling, i.e. the treatment) had no influence on the copulation duration in the second copulation in both sexes (linear model fits, females: F_2,69_ = 1.70, p = 0.19; males: F_2,56_ = 0.96, p = 0.49).

*Within-individual effects of mating status*

We analysed the subsets of individuals that were successfully mated twice to directly model the within-subject response to mating status. Female non-virgin copulations were on average 27.1 (95% CI: 15.7–38.5) minutes longer compared to corresponding virgin copulations (paired t test: t_71_ = 4.73, p < 0.001). Likewise, male non-virgin copulations were on average 27.0 (95% CI: 16.9–37.1) minutes longer than corresponding virgin copulations paired t test: t_59_ = 5.35, p < 0.001, Table 2).

*Sex-specific repeatabilities of copulation duration*

*Note: Here we used a fixed effect called CopType with four levels to consider the different mating combinations (e.g. virgin with non-virgin).*

*Sex-specific repeatabilities of copulation duration*

Repeatabilities for copulation duration amounted to 0.34 (95% CI: 0.10 – 0.55, LRT: χ^2^ = 8.65, df = 1, p = 0.003) for males and to 0.10 (95% CI: 0.00 – 0.30, LRT: χ^2^ = 0.75, df = 1, p = 0.39) for females.

**Table 1: Linear mixed effect model fits for copulation duration of *G. rufus* treating relatedness with two levels and mating status with four levels as two factorial predictors.**

*Note: Here we used a fixed effect called CopType with four levels to consider the different mating combinations (e.g. virgin with non-virgin).*

| Fixed effects: | Estimate | SE | χ² | *P* |
| --- | --- | --- | --- | --- |
| Intercept | 119.06 | 4.36 |  |  |
| Sibling | -4.05 | 4.62 | 0.78 | 0.38 |
| Cop. Type Non-v./Virgin | -28.28 | 10.27 |  |  |
| Cop. Type Virgin/Non-v. | -0.48 | 22.26 | 21.61 | <0.001 |
| Cop. Type Virgin/Virgin | -26.02 | 4.27 |  |  |
|  |  |  |  |  |
| Random effects: | Variance | SD | χ² | *P* |
| Female ID (Intercept) | 93.61 | 9.68 | 0.75 | 0.39 |
| Male ID (Intercept) | 371.80 | 19.28 | 8.65 | 0.003 |

**Table 2: Summary statistics for copulation durations (min) separated by sex and copulation type (regardless of mating partner relatedness) and paired t-test testing for differences between mating status within sexes.**

| Sex | Copulation type | Mean ± SD | Range | *N* | *P* |
| --- | --- | --- | --- | --- | --- |
| Females | Virgin | 84.95 ± 29.6 | 17 – 169 | 72 | \|<0.001 |
|  | non-virgin | 116.7 ± 40.1 | 14 – 213 |  |  |
| Males | Virgin | 88.37 ± 30.1 | 17 – 169 | 59 | \|<0.001 |
|  | non-virgin | 115.3 ± 38.4 | 26 – 213 |  |  |
